# Long COVID Syndrome: A Narrative Review on Burden of Age and Vaccination

**DOI:** 10.3390/jcm13164756

**Published:** 2024-08-13

**Authors:** Panaiotis Finamore, Elena Arena, Domenica Lupoi, Luisa Savito, Francesca Di Nunzio, Michela Furbatto, Silvano Dragonieri, Raffaele Antonelli Incalzi, Simone Scarlata

**Affiliations:** 1 Unit of Internal Medicine, Fondazione Policlinico Universitario Campus Bio Medico, 00128 Rome, Italy; p.finamore@policlinicocampus.it (P.F.); elena.arena@unicampus.it (E.A.); domenica.lupoi@unicampus.it (D.L.); luisa.savito@unicampus.it (L.S.); francesca.dinunzio@unicampus.it (F.D.N.); michela.furbatto@unicampus.it (M.F.);; 2 Research Unit of Internal Medicine, Università Campus Bio-Medico, 00128 Rome, Italy; 3 Department of Respiratory Diseases, University of Bari, 70124 Bari, Italy; silvano.dragonieri@uniba.it

**Keywords:** COVID-19, vaccine, aging, long COVID

## Abstract

**Background/Objective:** The COVID-19 pandemic has led to the emergence of post-acute COVID-19 syndrome, also known as long COVID, which presents a significant challenge due to its varied symptoms and unpredictable course, particularly in older adults. Similar to COVID-19 infections, factors such as age, pre-existing health conditions, and vaccination status may influence the occurrence and severity of long COVID. The objective is to analyze the role of aging in the context of long COVID and to investigate prevalence rates and vaccination efficacy to improve prevention strategies and treatment in this age group. **Methods:** Four researchers independently conducted a literature search of the PubMed database to trace studies published between July 2020 and July 2024. **Results:** Aging influences both the likelihood of developing long COVID and the recovery process, due to age-related physiological changes, immune system alterations, and the presence of comorbidities. Vaccination plays a key role in reducing the risk of long COVID by attenuating the inflammatory responses associated with its symptoms. **Conclusions:** Despite the protection vaccines offer against severe infection, hospitalization, and post-infection sequelae, vaccine hesitancy remains a major obstacle, worsening the impact of long COVID. Promising treatments for this condition include antivirals although further research is needed.

## 1. Introduction

The advent of the novel severe acute respiratory syndrome coronavirus-2 (SARS-CoV-2), first reported in Wuhan, China, in December 2019 and responsible for the global pandemic a few months later, marked an unprecedented global health crisis in modern times. With its rapid spread, the virus has since claimed almost 7 million lives as of December 2023 [1]. Although the acute phase of the pandemic is no longer classified as a global emergency, a growing number of individuals are still experiencing persistent and often debilitating symptoms, known as “long COVID” or “post-acute sequelae of SARS-CoV-2 infection (PASC)”, defined by the WHO as “a condition that occurs in individuals with a history of probable or confirmed SARS-CoV-2 infection, usually 3 months from the onset, with symptoms that last for at least 2 months and cannot be explained by an alternative diagnosis” [2]. Long COVID was initially reported in July 2020 by Carfi et al. [3] and was later investigated by several meta-analyses, suggesting that approximately 30–50% of individuals may experience enduring symptoms lasting up to a year [4,5], while a more recent meta-analysis concluded that the global prevalence of long COVID after two years from the infection is estimated to be approximately 43% [6].

Long COVID unfolds as a complex and multifaceted phenomenon, with an extensive spectrum of over 100 manifestations [7], more commonly fatigue, headache, attention disorders, hair loss, and dyspnea [8], distributed heterogeneously across age groups. Specifically, older people frequently experience mood disorders, fatigue, and anxiety [9]. Conversely, in another study, the most reported symptoms consisted of fatigue, breathlessness, joint pain, and cough [10]. It is worth highlighting that the available evidence concerning older populations aged >75 years old is limited and inconclusive, emphasizing the need for more comprehensive investigations regarding this demographic.

Understanding the interplay between age and long COVID is essential for several reasons. Firstly, it enables the identification of vulnerable populations, facilitating targeted interventions. Secondly, insights into age-specific manifestations contribute to our understanding of the underlying mechanisms of long COVID, guiding therapeutic strategies. Lastly, recognizing age-related patterns aids in accurate prognostication, allowing healthcare professionals to anticipate and address potential complications in a more nuanced manner.

The advent of COVID-19 vaccines has added a powerful tool to the arsenal against long COVID. Beyond mitigating the risk of severe acute infection, vaccination appears to reduce the likelihood of developing persistent symptoms [11,12,13]. In addition, one meta-analysis suggests that a large number of individuals reported improvement in pre-existing post-COVID symptoms following vaccination [14]. Investigating the impact of vaccination across different age groups is crucial for optimizing immunization strategies. Furthermore, understanding the intricate relationship between age, vaccine effectiveness, and long COVID can inform public health efforts, guiding the prioritization of specific age cohorts for vaccination campaigns.

The aim of this narrative review is to examine how age and vaccination intersect in the context of long COVID, particularly among older adults. By investigating prevalence rates and vaccination efficacy, the study seeks to provide tailored insights for managing long COVID in this age group effectively.

## 2. Methods

A literature search of the PubMed database was conducted by four independent authors (E.A., M.F., D.L., L.S.) to trace all relevant studies published between July 2020 and July 2024.

The search strategy consisted of using combinations of the following terms: “Long COVID AND older adults”; “Long COVID AND survival”; “Long COVID AND vaccination”; “Long COVID AND treatment”.

**Figure 1:**
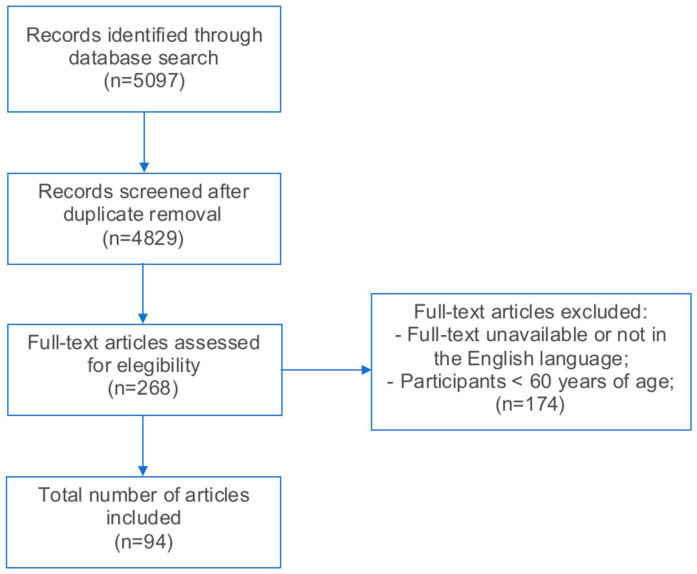
PRISMA diagram.

Full-text articles in the English language evaluating long COVID effects in older individuals (>60 years of age) were included, with no exclusion criteria. Ninety-four articles met our inclusion criteria and were included in this review (Figure 1).

### 2.1. Age as a Risk Factor for Long COVID

Age is a critical determinant of the risk and outcomes associated with long COVID. Older adults, typically more susceptible to severe acute infection, often face prolonged and complicated recovery periods, especially if unvaccinated [15,16]. The impact of aging on long COVID is multifaceted, involving physiological, immunological, and psychological factors [17].

Physiologically, aging contributes to a decline in organ function and regenerative capacity, impacting the body’s ability to recover from the acute phase of infection. Older individuals may experience a more protracted course of illness, with persistent symptoms affecting various organ systems. Age-related changes in lung function, cardiovascular health, and neurological resilience may all contribute to the prolonged and diverse symptoms observed in long COVID patients [18,19].

With advancing age, the respiratory system undergoes structural and functional alterations, which include decreased lung elasticity, resulting in reduced lung compliance and impaired gas exchange, weakened respiratory muscles, and impaired mucociliary clearance. These factors collectively contribute to the decline in lung function and increased susceptibility to respiratory disorders in older adults [20].

Furthermore, aging is associated with an increased prevalence of cardiovascular conditions such as hypertension, coronary artery disease, and heart failure. Among the physiological changes, a notable one is arterial stiffness, which results in increased vascular resistance and elevated blood pressure. Similarly, myocardial fibrosis induces left ventricular hypertrophy and impaired global cardiac function. The decline in baroreflex sensitivity and autonomic function further impacts cardiovascular health by disrupting blood pressure regulation and cardiac electrical activity, including prolongation of the QT interval and alterations in atrial and ventricular conduction [21].

The aging process affects the structure and function of the nervous system and encompasses both gross and microscopic structural alterations, including cerebral atrophy, lipofuscin accumulation, and the presence of neurotoxic proteins like amyloid-beta and neurofibrillary tangles. Biochemical and metabolic changes involve disruptions in neurotransmission molecules and imbalances in neurotransmitter levels, affecting various brain regions and leading to dysfunction. Cellular and molecular changes involve DNA expression alterations, mitochondrial dysfunction, and impaired clearance systems. Aging is also associated with changes in the peripheral nervous system, including a decline in nerve conduction velocity and sensory function [22].

One of the key contributors to the age-related vulnerability to long COVID is the change in immune function. The immune system undergoes alterations with age, collectively known as “immune-senescence”, first coined by R. Walford [23], which is characterized by a reduced or impaired function of innate and adaptive immunity, therefore affecting the body’s ability to mount an effective response against pathogens.

Older individuals may exhibit decreased T-cell function, impaired cell-mediated and adaptive immune responses [23], and chronic low-grade systemic inflammation, known as inflammaging [24], all of which contribute to the chronic nature of long COVID symptoms [25]. A principal component analysis performed on 19 inflammatory biomarkers in the “InCHIANTI” study revealed that cytokines involved in inflammaging, such as IL-1 receptor antagonists, IL-6 and TNF-alfa, are responsible for the COVID-19 cytokine storm and long COVID [26].

Research suggests that age-related differences in the composition of the immune system may lead to a prolonged and dysregulated immune response in long COVID patients [24]. This dysregulation may contribute to the persistence of symptoms and the development of conditions such as brain fog, fatigue, and respiratory issues. Recognizing these age- and sex-specific immunological challenges [27] is crucial for developing targeted therapies and interventions tailored to different age groups.

**Figure 2:**
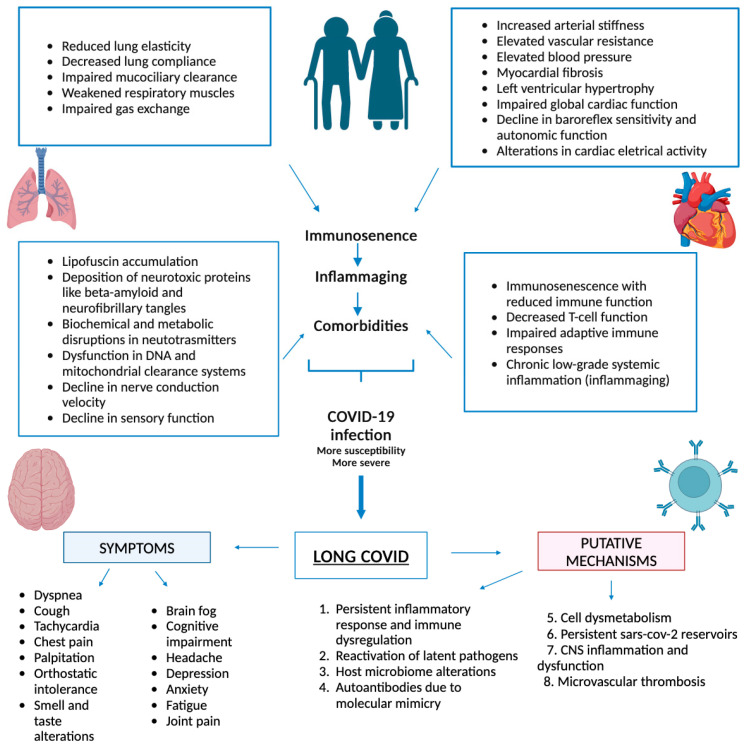
Age-related changes in the function of various organ systems and their role in the pathophysiology and clinical expression of long COVID.

In conclusion, age emerges as a critical factor in comprehending the complexity of long COVID (Figure 2). It influences both the risk of developing persistent symptoms and the trajectory of recovery [28]. Recognizing age-specific vulnerabilities and leveraging vaccination strategies tailored to different age groups can significantly mitigate the impact of long COVID. A comprehensive understanding of the interplay between age and long COVID is essential for developing targeted interventions and promoting long-term public health. The integration of age-specific considerations into research, clinical practice, and public health initiatives will pave the way for more effective strategies to address the multifaceted nature of long COVID across diverse age groups.

### 2.2. Geriatric Syndromes and Long COVID

There exists a strong correlation between frailty, in the sense of poor ability to respond to stress due to the aging of the organism, and the risk of complications caused by COVID-19, including long COVID; this is witnessed by the great number of deaths and long-term complications in Long-Term Care Facilities all over the world during the COVID-19 pandemic. In fact, in these realities, the population is represented by individuals with many chronic conditions [29]. Regarding long COVID, a systematic review and meta-analysis of 120,970 patients published in 2022 showed that older age represents an important risk factor for developing it, with general, psychiatric, respiratory, digestive, and skin signs and symptoms, but particularly mobility issues, which frequently precede disability [30]. Even Wander et al., in their retrospective cohort study, have demonstrated that long COVID was documented with major frequency among older people with a high burden of comorbidities (for example, COPD, diabetes, CKD, depression, and post-traumatic stress disorder [31]. It is likely that the presence of multiple chronic conditions (i.e., multimorbidity) is one of the factors most associated with the risk of documented long COVID care, as measured by the Charlson Comorbidity Index score by George N. Ioannou et al. [32]. Age, frailty, and comorbidities explain why older adults are more susceptible to persistent symptoms than younger individuals, who recover more quickly from the acute phase of infection [28].

### 2.3. Epidemiological Insights and Clinical Profile

According to estimates from the Centers for Disease Control and Prevention (CDC), about one in four COVID-19 survivors aged ≥65 years experienced at least one incident condition that might be attributable to a previous infection [33], with the most commonly reported symptoms being fatigue, shortness of breath, and cognitive dysfunction, with patients still experiencing symptoms after 3 months, as reported by the European Observatory on Health Systems and Policies [34]. A retrospective cohort study [35] found an increased risk [risk differences of 2.39 (95% Cl—1.79 to 2.94)] of one or more new or persistent clinical sequelae after COVID-19 infection in adults older than 65 years, compared with two historical comparison groups. Disparities in long-COVID burden across different age groups have been observed, with older adults experiencing a higher incidence of chronic conditions and a significant limitation in their daily activities due to ongoing symptoms related to COVID-19 [36]. COVID-19 has also been shown to exacerbate chronic conditions that occur commonly in older people, such as cardiovascular diseases, respiratory diseases, neurodegenerative conditions, and functional decline [37]. A case-controlled veteran cohort study with a mean age of 63 [38] found that people who had COVID-19 showed increased risks and 12-month burdens of cardiovascular disease, including cerebrovascular disorders, dysrhythmias, inflammatory heart disease, ischemic heart disease, heart failure, thromboembolic disease, and other cardiac disorders, with the risks evident independent of age. Another case-controlled study based in Sweden [39] found that acute COVID-19 infection increased the risk of subsequent deep vein thrombosis, pulmonary embolism, and bleeding, with a higher risk in older age groups (>50 years old) than in younger age groups. Moreover, a comparison of long COVID outcomes between younger and older individuals has shown that older adults are more likely to experience severe and persistent symptoms, leading to a greater impact on their overall health and well-being, as shown in a recent meta-analysis of observational studies [40]. Data also suggests a fair variability in expected recovery time and prognosis, mainly depending on older age, pre-existing risk factors, and acute COVID-19 infection severity [41]. The main available evidence on this topic is summarized in Table 1.

In a 2020 study [28], hospitalized patients aged >50 years old were more likely to experience residual symptoms than younger people aged between 18 and 35 years (47% vs. 26%). Otherwise, young, healthy patients with mild disease also recovered more rapidly than older patients or those with multiple co-morbidities, although 26% of those aged 18–35 years still experienced prolonged symptoms.

Clinical features of long COVID in the older population are shown in Table 2.

### 2.4. Role of Pharmacological and Non-Pharmacological Therapies in Mitigating Long COVID in Older Adults

Tailored management for long COVID encompasses an understanding of age-related factors. A multidisciplinary approach has shown promise for improving symptoms and physical capacity in long COVID patients, including older adults. A 2023 study showed that a targeted multidisciplinary rehabilitation program improved body composition, dyspnea, fatigue, and physical capacity in long COVID patients [44]. These programs should be aimed at specific needs related to age-related comorbidities and functional and cognitive decline, helping older adults regain their independence and quality of life; however, data on this subpopulation is still lacking. The impact of age on the response to various treatment modalities and interventions is an important consideration in the management of long COVID in older adults. Evidence on antiviral treatment’s effect administered during acute COVID-19 infection and its impact on reducing long-term symptoms is still scarce. Moreover, the response to various treatments and interventions in the elderly may be reduced due to age-related changes in the body’s ability to process and respond to treatments; therefore, trials on this specific subpopulation are warranted. Recently, a small reduction in post-COVID conditions was found among US adults ages 65 and older who were treated with either the antiviral drug nirmatrelvir or molnupiravir [45]. Another single-center Italian study based on hospitalized patients with COVID-19 with a median age of 65 years suggested that treatment with remdesivir during acute illness was associated with a lower risk of developing long COVID compared to no antiviral treatment [46]. However, medication management in the elderly involves careful selection and dosage adjustments to accommodate age-related changes in metabolism and organ function. Further research is warranted on this topic. Moreover, the mental and social effects of the COVID-19 pandemic itself should not be underestimated [37]. The results of social deprivation could be profound and lead to cognitive and functional decline, especially for those living in aged care facilities [42]. As the number of people infected with SARS-CoV-2 increases, the number of survivors suffering post-COVID conditions is also likely to increase. Epidemiological studies have highlighted the disproportionate burden faced by older adults, and tailored management approaches are essential to address the specific needs of this population. Further research is needed to identify effective treatment options and interventions for older adults with long COVID.

### 2.5. Role of Vaccination in Mitigating Long COVID in Older Adults

In addition to being effective against COVID-19 disease, both symptomatic and severe, COVID-19 vaccines appear to be active in preventing long COVID. In this regard, a recent systematic review and meta-analysis showed that vaccination is particularly useful in reducing the risk of long COVID but only in those who received two doses, independently of a previous SARS-CoV-2 infection [13]. Ceban and colleagues, in their systematic review and meta-analysis, on the other hand, demonstrated that at least one dose of a COVID-19 vaccine may be protective against the development of long COVID breakthrough infections [14]. This response after vaccination seems to be related to the attenuating action of the vaccine against the pro-inflammatory responses associated with long COVID symptoms by decreasing the levels of certain cytokines and chemokines, but also the intensity of the acute phase immune response [47]. Regarding older age, numerous studies have shown an important reduction in the risk of long COVID among the elderly (age > 60 years) vaccinated with at least two doses [37]. Comparing the efficacy of different vaccines, a cohort study of data from the UK, Spain, and Estonia showed that BNT162b2 had a slightly higher vaccine effectiveness in preventing persisting COVID-19 symptoms than ChAdOx1, probably because people might have been less likely to be infected with SARS-CoV-2 in the first place [48]. Currently, all approved vaccines have shown strong efficacy, demonstrating induction of antibody levels of similar or higher magnitude as those observed in convalescent individuals and near-complete protection against hospitalization and severe disease [49].

Vaccination plays an important role in reducing long COVID symptoms. Several studies, conducted in different countries and across different age demographics, have demonstrated that vaccination-induced immunity is able to protect the individual from long COVID, although with different success rates [50,51,52]. Nayyerabadi and colleagues have suggested that the protective power of the vaccine rests on the fact that it could mitigate inflammatory reactions by determining a reduction of cytokines such as IL-6, IL-2, IL-8, TNF-α, and interferons independently of the number of doses received [47]. The COVID-19 vaccine has also had an impact on plasma sCD40L levels; in fact, they were significantly reduced following vaccination; plasma sCD40L levels are typically high in moderate to severe COVID-19, and high levels have also been reported in conditions that predispose patients infected with SARS-CoV-2 to develop long COVID, such as hypertension [53]. Regarding the innate immune response, a recent study has shown how plasma levels of MIP-1α, IL-12p40, G-CSF, M-CSF, SCF, and IL-1β were elevated in participants with long COVID pre-vaccination, reflecting inflammatory chronicity and altered immune competence that could be restored by vaccination, but this aspect warrants further investigation [47,48,49,50,51,52,53,54]. Failure to protect a share of patients from long COVID may be due to persistently circulating viral components despite vaccination, evidence of the presence of a viral reservoir that may not be cleared following one or two doses of vaccination, and may contribute to sustaining the inflammatory response. Regarding SARS-CoV-2 reactive immunoglobulins, Nayyerabadi and colleagues observed that participants with long COVID had increased levels of IgG reactive to SARS-CoV-2 RBD and spike proteins after receiving one or two vaccine doses but decreased levels of IgM, to emphasize the fact that patients with long COVID retain a certain level of sustained immunoreactivity toward SARS-CoV-2, which is boosted in response to mRNA vaccination [22].

The chief weapon to be used in containing the COVID-19 pandemic remains vaccination, in all age demographics. COVID-19 proceeds more softly in children and adolescents than in adults; however, the vaccination is still indicated considering the risk of all the acute effects and sequelae of SARS-CoV-2, including the risk of the multisystem inflammatory syndrome in children, indirect effects on mental health and education, and also long COVID. Regarding adults and older people, several observational studies have shown that breakthrough infection in vaccinated individuals is associated with fewer and shorter durations of symptoms, a lower probability of long COVID, and a higher likelihood of asymptomatic infection compared with infection in unvaccinated individuals. In this context, adults older than 65 years represent a special population because, in this age group, COVID-19 might lead to an increased health burden, both directly from specific long COVID-associated symptoms and from the exacerbation of pre-existing health issues, such as diabetes and cardiovascular disease. For these reasons, it is crucial for older people (especially those living in aged care facilities) to get involved in vaccination campaigns in order to ensure that this population is not deprived of the benefits of multidisciplinary care that can improve their functioning and quality of life [37]. A recent article about vaccination strategies to mitigate the impact of SARS-CoV-2 transmission has demonstrated the importance of continued booster doses as part of the wider public health response to ongoing endemic transmission of SARS-CoV-2, giving priority to the elderly; this could represent a strong strategy in order to reduce SARS-CoV-2 sequels, hospitalizations, and death [55].

### 2.6. Vaccine Hesitancy and Acceptance in COVID and Long COVID Outbreak—Socio-Economic Implications and Vaccination Campaigns

Vaccine hesitancy remains a critical concern amidst the global effort to combat the COVID-19 pandemic. While the World Health Assembly emphasized the significance of widespread immunization, as of July 2024, only 70.6% of the global population has received at least one vaccine dose [4]. This hesitancy poses challenges to achieving herd immunity. Understanding vaccine acceptance and hesitancy is vital for effective public health strategies.

Research has shown varying degrees of hesitancy across age cohorts, influenced by factors such as perceived risk, confidence in vaccines, misinformation, and accessibility [55,56,57,58,59,60,61,62]. Studies demonstrate a spectrum of attitudes towards the COVID-19 vaccination, with some showing resistance among young adults and greater acceptance among the elderly. Factors such as gender, fear of COVID-19, and trust in healthcare institutions also influence vaccine acceptance [58,59,60].

Addressing vaccine hesitancy requires a multifaceted approach, considering demographic, social, and psychological factors in order to combat misinformation and increase trust in vaccines and healthcare systems [62,63,64]. Additionally, tailored interventions targeting specific age groups and communities can enhance vaccine acceptance and uptake.

The prevalence of long COVID further complicates the issue, with vaccination status impacting the likelihood of experiencing persistent symptoms. Research suggests that vaccination reduces not only the severity and duration of COVID-19 symptoms but also the risk of long COVID, highlighting the importance of vaccination in preventing post-infection complications [65,66,67,68,69,70].

A synopsis of available evidence on the role of vaccination in long COVID is reported in Table 3.

Identified risk factors for long COVID include gender, age, BMI, comorbidities, smoking, and prior hospitalization. Yet, the consequences of non-vaccination on long COVID remain ambiguous [71]. Studies highlight a disparity in long COVID prevalence between vaccinated and unvaccinated individuals, as well as variations based on vaccination timing and dose numbers [65,66,67,70].

Some research indicates that vaccination prior to SARS-CoV-2 infection reduces long COVID prevalence and symptom severity [65]. Conversely, other studies suggest a decrease in long COVID prevalence even in individuals vaccinated post-infection [67]. Notably, receiving two vaccine doses appears to be more effective in preventing long COVID compared to a single dose, with protection increasing with additional doses [65,72]. In addition to preventing initial SARS-CoV-2 infection, vaccination may mitigate long COVID risk through two proposed mechanisms: reducing initial infection severity and accelerating viral clearance, or mitigating excessive inflammatory responses. Evidence suggests survivors of COVID-19 exhibit elevated antinuclear antibodies associated with prolonged COVID symptoms, supporting the hypothesis that vaccines could aid in symptom reduction [73].

Further research is essential to elucidate vaccination’s role in long COVID prevention and management and inform public health strategies to combat the ongoing pandemic effectively.

Moreover, long COVID represents a health issue for the individual and a huge burden for the entire community. The long-term economic burden of a substantially large long COVID population will emerge over time and is expected to have a strong impact on healthcare utilization costs [74]. According to an issue of The Lancet Regional Health—Europe, between 11 and 52% of workers with long COVID may not return to work 6–12 months after the infection [75], with an overall impairment of quality of life, social and family life, and level of functioning. There are therefore both health and socio-economic reasons why it is important to combat vaccine hesitancy to reduce the impact of long COVID.

**Table 3:** Vaccination and long COVID, hesitancy, and acceptance.

First Author/Year	Study Design Sample Size	Population Outcome	Main Finding
Gao/2022 [13]	Systematic Review and Meta-Analysis	Relationship between vaccination and long COVID	Two doses of vaccine (but not a single one) protect against the symptoms of long COVID, particularly those related to concentration, memory, smell, taste, breathing, asthenia, and headache. A similar result was obtained with vaccination even after COVID, as it appears to increase viral clearance and immune response.
Ceban/2023 [14]	Systematic Review and Meta-Analysis	Identify the risk of long COVID among vaccinated and unvaccinated individuals and the trend of long COVID following the vaccination	Vaccination protects against long COVID symptoms when administered before infection and reduces them when already present, with variable responses depending on the genetic, environmental, and behavioral factors of each patient.
Mansell/2022 [37]	Personal View	Relationship between long COVID, comorbidities, and fragility in elderly patients	Elderly patients, defined in this study as aged 65 or older, have a higher likelihood of experiencing long COVID symptoms. Particularly, older adults residing in long-term care facilities are more exposed to the risk of clinical, social, and psychological alterations. Moreover, some long COVID symptoms, such as fatigue, could be confused with frailty symptoms, highlighting the need for special attention to this patient population, in which vaccination of at least two doses appears to reduce the symptoms of long COVID.
Català/2024 [48]	Staggered Cohort Study	Overall effect of vaccination in preventing long COVID and comparison of efficacy between ChAdOx1 e BNT162b2. HRs of COVID-19 infection in people receiving two doses of BNT162b2 ranged from 0.65 (95% 0.59–0.71) for females to 0.80 (95% 0.70–0.90) for oldest-old adults.	This cohort study involved more than 20 million people aged between 26 and 84 years residing in Spain, the United Kingdom, and Estonia. A comparative analysis between BNT162b2 and ChAdOx1 showed a greater protective effect of the first one against long COVID. Vaccination, with slight differences between 1 and 2 doses, was able to protect up to 52%.
Al-Aly/2022 [51]	Observational study	Compared to people with COVID-19 who were not previously vaccinated (*n* = 113,474), people with BTI exhibited lower risks of death (HR = 0.66, 95% CI: 0.58, 0.74) and incident post-acute sequelae (HR = 0.85, 95% CI: 0.82, 0.89).	Among the analyses of this study, 33,940 patients with BTI were compared to 113.474 unvaccinated patients infected with SARS-CoV-2. A decreased risk of long COVID among individuals vaccinated prior to SARS-CoV-2 infection was observed (HR 0.85) compared to those who were unvaccinated, with a reduction of 24 long COVID sequela out of 47 examined. The reduction in sequela was observed in subjects vaccinated with BNT162b2 and mRNA-1273 compared to those vaccinated with Ad26.COV2.S. However, the study highlights how other preventive measures, beyond vaccination, remain essential.
Azzolini/2022 [52]	Observational Cohort Study	Compare vaccinated individuals with mRNA vaccines to unvaccinated individuals regarding the risk of long COVID.	This observational cohort study involved 2560 non-hospitalized healthcare workers who were asked to receive 3 doses of BNT162b2 vaccine. It was observed that the incidence of long COVID decreased with an increase in vaccine doses. Specifically, long COVID occurred in 41.8% of the unvaccinated, 30% of those vaccinated with one dose, 17.4% of those vaccinated with 2 doses, and 16% of those vaccinated with 3 doses. Time between the second vaccination dose and infection was not associated with long COVID (OR, 0.66; 95% CI, 0.34–1.29).
Callaghan/2021 [56]	Survey	Provide an overview of the U.S. population regarding COVID vaccination	Vaccine hesitancy, in this survey, appears to be correlated with various factors, including gender, race, income, and political views. Specifically, the data obtained showed that individuals intending to vote Trump in the 2020 presidential elections, were less inclined to consider receiving a COVID-19 vaccine once it became available.
Solìs Arce/2021 [57]	Survey	Relationship between vaccine acceptance and the country’s income	This study involved 44,260 individuals residing in 10 low- and middle-income countries, as well as the USA and Russia. Categorizing participants into three age groups (<25, 25–54, and >55), it emerged that the most common motivation for accepting the vaccine was self-protection across all age groups. However, the decision to get vaccinated motivated by a sense of protection towards one’s family and the community was noted in relation to age groups and geographical location. The findings of this study suggest that age is likely interconnected with other factors, including income.
Lin/2020 [58]	Systematic Review	Identify the state of vaccine acceptance based on U.S. and international surveys	This systematic review included 126 surveys conducted in 31 countries, mainly in the USA. It was shown that vaccine hesitancy depends on numerous factors, including age (with younger individuals being more inclined to vaccination in some surveys, while in others, adults over 55 were), gender (generally women are more hesitant), as well as other demographic characteristics, ethnicity, income, perceived risk of infection, trust in vaccine characteristics, transparency in their creation and distribution, and trust in healthcare workers and the government.
Roozenbeek/2020 [59]	Survey	Susceptibility to misinformation and the development of false beliefs and their role in adopting health behaviors	This study evaluates the role of misinformation on vaccine hesitancy in 5 countries: Ireland, the USA, Spain, Mexico, and the United Kingdom. It emerged that although the majority of the population could defend themselves against misinformation, it still represents a widespread phenomenon capable of undermining vaccination campaigns. It was noted that, for all countries except Mexico, older age was less susceptible to misinformation and that, in general, trust in scientists was a protective factor.
Roberts/2022 [60]	Survey	Predictors of vaccine hesitancy	This study, conducted among 1004 adults, found that younger age was associated with increased vaccine hesitancy and that the problematic use of social media also directly influences the adoption of anti-vax attitudes. Moreover, if an individual doesn’t believe that COVID-19 vaccines and other infectious disease vaccines are necessary and important, they may not be motivated to engage in related health and safety behaviors.
Antonelli/2022 [61]	Case-control Observational Study	Relative likelihood of developing long COVID after infection with the Delta variant versus the Omicron variant	This case-control observational study conducted in the United Kingdom on nearly 100,000 adults, showed that the risk of long COVID is more common after infection with the SARS-CoV-2 Delta variant (10.8%) compared to the Omicron variant (4.5%), with variables of age and time since vaccination.
Babicki/2023 [62]	Retrospective study	Investigate the manifestations of long COVID in relation to vaccination status	This retrospective study involving 801 participants, both vaccinated and unvaccinated, showed no discernible differences in long COVID diagnosis between the two groups. However, upon examining individual symptoms, it was revealed that headaches, joint pains, and blood pressure irregularities were notably more prevalent among unvaccinated individuals.
Czajka/2020 [65]	Survey	Determine the factors that most influence attitudes toward vaccines and decisions regarding them	This study conducted among 6432 adults on vaccine hesitancy in Poland identified the significant role of healthcare professionals in vaccine communication and debunking myths, showing how demographic variables have different effects on vaccine hesitancy.
Fisher/2021 [67]	Survey	Understand how race/ethnicity can influence parents’ decisions about vaccinating their children	This study, conducted on 400 mothers of children aged between 5 and 10 years, shows how race, along with other demographic factors such as education and income, significantly influences vaccine hesitancy. It demonstrates that the non-Hispanic white population is particularly resistant to vaccinating their children, regardless of the child’s age or gender. These findings may be important to consider when implementing public health measures.
Tsampaisan/2023 [64]	Systematic Review and Meta-Analysis	Identify demographic factors and comorbidities that could increase the risk of long COVID	This systematic review and meta-analysis were conducted among 41 studies on 870,783 patients, has shown that long COVID is a multifactorial syndrome in which female gender, age over 40 years, higher BMI, pre-existing comorbidities, smoking, previous hospitalization, and admission to an intensive care unit, influence the incidence of long COVID. Furthermore, this study highlighted that vaccination with two doses reduces the risk of long COVID compared to non-vaccination.
Williams/2021 [71]	Opinion	Proposal for vaccine implementation based on lessons learned from the past	This study was conducted to highlight the challenges in vaccine distribution in Sub-Saharan Africa. Vaccines should be affordable even for low- and middle-income countries. A strong national policy capable of overseeing the vaccination process and ensuring timely procurement and availability of resources, while also monitoring adverse effects to minimize risks, is essential. A robust post-marketing surveillance is necessary to characterize the risk profile in order to minimize it. Adequate health information systems are necessary for documenting and managing data to monitor progress, identify challenges, and provide evidence for administrators and policymakers. Effective communication about the benefits of the vaccine is of fundamental importance.
Bokemper/2021 [72]	Randomized survey experimented	The influence of politics on vaccine acceptance	This study, based on two experiments conducted in the USA involving 5014 people, highlighted how vaccine acceptability is influenced by the timing of its distribution and the political context. Moreover, there are high levels of trust in healthcare workers, suggesting that communication strategies for social and behavioral change involving local healthcare workers can be particularly effective in combating residual hesitancy.
Fadda/2020 [73]	Letter	Challenges that the COVID-19 vaccine needed to address	This study was conducted before the advent of vaccines for SARS-CoV-2: there were anticipations that the swift pace of vaccine development could amplify public apprehensions, potentially impacting their readiness to embrace the vaccine. Therefore, it is essential to train healthcare providers and other key stakeholders in the immunization landscape in effective communication with individuals.
Abdullahi/2023 [76]	Cross-Sectional Study	The planning of mass vaccination in Qatar and GCC countries	Like in Kuwait, Oman, and Saudi Arabia, the prioritization grouping in Qatar gave priority to vaccination for three categories of risk: key workers (such as teachers, healthcare workers, and essential government personnel), clinically vulnerable populations other than the elderly, and the elderly. However, unlike other GCC countries, the prioritization grouping in Qatar was quickly expanded to include 17 priority groups for vaccine administration.

It should be noted that vaccine hesitancy towards COVID-19 vaccines varies significantly across countries and demographic groups, posing a formidable challenge to vaccination campaigns [77]. Understanding effective vaccination strategies is crucial to reducing COVID-19 incidence and long COVID prevalence. Analysis of successful vaccination campaigns worldwide provides valuable insights for future implementations. The main European countries, for example, in their immunization COVID-19 campaign for the 2023/2024 season, recommended vaccination only for specific subgroups of people, taking into account various risk factors such as age, diseases, or frailty [78]. Notably, Portugal’s multifaceted approach, led by healthcare workers and supported by governmental and non-governmental organizations, exemplifies the effective dissemination of vaccine information and widespread acceptance. Similarly, Cuba’s self-reliant vaccine production and inclusive vaccination efforts, driven by national pride and community engagement, showcase the importance of awareness and self-determination [79].

In North America, the vaccination campaign among American Indians and Alaska Natives underscores the significance of culturally sensitive approaches tailored to the population’s needs and beliefs. Meanwhile, in high-income countries like Qatar, strategic prioritization and continuous improvement of vaccination processes have yielded substantial success, emphasizing the importance of adaptability and proactive measures [76].

Physicians’ collective involvement in vaccination advocacy emerges as a potent force, transcending individual expertise to mobilize broad organizational efforts and public confidence [80]. Effective communication strategies must consider community-specific literacy levels, cultural nuances, and concerns to foster trust and engagement. Cuba’s approach highlights the importance of recognizing and leveraging community strengths and limitations for optimal campaign outcomes.

Furthermore, harnessing the potential of social media as a knowledge dissemination tool is essential, emphasizing accurate information dissemination and global connectivity. An effective vaccination campaign requires a bottom-up and top-down approach, bridging governmental directives with community-level initiatives to ensure widespread coverage and acceptance [80].

In conclusion, successful vaccination campaigns integrate diverse stakeholders, prioritize community engagement, and adapt strategies to cultural contexts and societal needs. By learning from global examples and embracing proactive communication and inclusivity, future campaigns can optimize effectiveness and foster public trust in vaccination efforts.

Despite the great speed in developing COVID-19 vaccines, thanks to which pandemic control was possible, the poorest countries have not had access to vaccination, contributing to the spread of the virus and increased mortality. That is why international cooperation has been necessary to successfully distribute the vaccines to all countries in the world to contain the pandemic, particularly with the COVAX initiative (COVID-19 Vaccines Global Access Facility), which has ensured a fair distribution of diagnostic tests, therapies, and vaccines. However, equitable access is still far from reality; in fact, the disparity in the total share of people vaccinated against COVID-19 between low-income and high-income countries remains large (more than 80% of the population in high-income nations compared with less than 10% of the population in low-income countries as of early 2022) [43,81]. Long-term control of existing and future variants of COVID-19 requires ongoing use and funding of COVID-19 vaccination [82]. According to David J. Hunter et al., the medium-to-long-term solution for less developed and smaller countries could be access to local or regional vaccine production capacity in order to acquire independence from richer countries. Vaccines against pandemic diseases must be a global public good, and the ability to manufacture them should be continuously supported and commended [83,84].

Nevertheless, COVID-19 infection might still occur despite vaccination; consequently, additional preventive strategies and measures are necessary: hand washing and respiratory hygiene (e.g., covering the cough or sneeze) or alternatively, use of disinfectants containing at least 60% alcohol in the absence of visible dirt; adequate ventilation of indoor spaces; in case of typical symptoms, getting tested for SARS-CoV2 and staying home; avoiding close contact with individuals who have or may have COVID-19; wearing masks. In conclusion, better policies and practices are needed to effectively address the issue of long COVID, including improved definitions and data collection worldwide.

## 3. Discussion

Currently, long COVID remains a syndrome without specific therapeutic options. For this reason, the prevention and treatment of this condition are urgent clinical priorities. The literature analyzed in this review highlights that advanced age is a significant risk factor for the development of long COVID and of debilitating symptoms affecting the nervous, cardiovascular, respiratory, and digestive systems, with serious consequences for the general health and well-being of this population.

As preventive options for long COVID, vaccines have important potential benefits. The most recent research demonstrates that the use of at least two doses of the SARS-CoV-2 vaccine, especially in the over-65 population, helps reduce the prevalence, duration, and severity of long COVID symptoms and decrease the risk of developing this condition by accelerating viral clearance and mitigating excessive inflammatory responses.

As far as antiviral treatment, Remdesivir, nirmatrelvir, and molnupiravir administered within five days from symptoms onset during the acute phase of COVID-19 may reduce the post-acute sequelae of SARS-CoV-2 infection in all age demographics, particularly among frail elderly population.

### Limitations

The limitations of this review include the selection of articles only from the PubMed database, excluding other sources. This choice might have led to the omission of additional relevant studies. Moreover, only full English text reviews were included, and articles published in preprint databases were excluded due to the lack of peer review. The quality of the included studies may vary, potentially affecting the overall strength of the evidence. Additionally, the analyzed studies are heterogeneous in terms of demographic age, type of vaccine administered, and antiviral treatment. Consequently, the results may not lead to a definitive conclusion. The strength of this review lies in the extensive time span selected for the analysis of articles, from July 2020 to July 2024. Current diagnostic and treatment options are poor, highlighting the need for a multitude of clinical trials to rigorously assess potential treatments targeting underlying biological mechanisms. Conducting thorough clinical trials is imperative to progress our understanding and improve the effectiveness of diagnostic and therapeutic approaches. Future research should investigate factors influencing the trend of long COVID symptoms. Additionally, there is a need to explore the timing of changes following SARS-CoV-2 vaccination and understand how various demographic subgroups of long COVID patients respond to SARS-CoV-2 vaccination (how cellular immune responses from different vaccines could variably affect long COVID conditions) to gain a better understanding of the dynamics of long COVID and optimize vaccination strategies for different patient profiles.

## 4. Conclusions

Long COVID remains a challenging condition that warrants further study. One certainty is that age plays a crucial role in determining the time of recovery, clinical outcomes, and the efficacy of interventions and targeted public health initiatives.

Vaccines proved to be the principal tool in preventing long COVID by reducing the symptoms, severity, and duration of SARS-CoV2 disease. Existing antiviral treatments administered during acute COVID-19 infection have yet to prove sufficient in reducing the incidence of long COVID. More trials are necessary to elucidate the pathophysiological mechanisms, test medications, and enhance prevention strategies.

## Figures and Tables

**Table 1:** Main available evidence on long COVID.

First Author/Year	Study Design/ Sample Size	Population Outcome	Main Findings
Sathyamurthy/2021 [9]	Prospective cohort study/279 older adults	Estimated prevalence, pattern, and functional outcomes of post-COVID-19 syndrome in hospitalized older adults	The prevalence of long COVID-19 syndrome in older adults is about 9.3%; the most common symptoms reported by older adults after 90 days following recovery were fatigue followed by cough and breathlessness.
Tosato/2021 [10]	Cross-sectional study/165 older adults	Prevalence of persistent symptoms among older COVID-19 survivors/(PR 2.67; 95% CI 1.01–10.6)	Persistence of COVID-19 is frequently experienced by older adults who have been hospitalized for COVID-19.
Calabrò/2023 [15]	Systematic Review/748 patients	The impact of vaccination on COVID-19 mortality	Fully vaccinated individuals have a lower risk of death, severe symptoms, and hospitalization compared to unvaccinated individuals.
Yang/2023 [16]	Systemic review and meta-analysis	Effects of vaccination on COVID-19-related mortality and complications	SARS-CoV-2 vaccines administered to the elderly are effective in preventing breakthrough infection, hospitalization, severity, and death.
Lee/2007 [17]	Original research/175 patients	Stress level and psychological distress of SARS survivors 1 year after the outbreak	SARS survivors have elevated stress levels and worrying levels of psychological distress.
Matsumoto/2023 [18]	Report		Possible influences and pathophysiological mechanisms of long COVID on hypertension-related organs, including the cardiovascular system, kidney, and endocrine system
Daitch/2022 [19]	Multicenter, prospective cohort study/2333 patients	Description of long-COVID symptoms among older adults and assessment of the risk factors for two common long-COVID symptoms: fatigue and dyspnea/(OR 0. 779, 95% CI 0.538–1.129)	Older individuals with long-COVID have different persisting symptoms, with more pronounced pulmonary impairment.
Schneider/2021 [20]	Review		Molecular and cellular aspects of lung aging, local stress response pathways, and how the aging process predisposes to the pathogenesis of pulmonary disease in the context of the COVID-19 pandemic.
Ferrari/2003 [21]	Invited review		The cardiovascular and reflex changes brought about by aging may have significant implications for circulatory homeostasis in health and disease
Nikolich-Žugich/2018 [23]	Review Article		Understanding of age-related changes that affect key components of immunity
Morrisette-Thomas/2014 [24]	Randomized Controlled Trial/1453 patients	Impact of biomarkers of inflammation in predicting chronic disease and aging/HR 1.33; 95% CI 1.16–1.53	Multidimensional approach allows a more robust interpretation of the various relationships between the pro-inflammatory biomarkers.
Müller/2023 [25]	Review		Immunosenescence and inflammation contribute to the emergence and progression of autoimmune disorders in the elderly and may serve as potential mediators for long COVID disturbances
Tenforde/2020 [28]	Report/292 patients	Impact of older age and presence of multiple chronic medical conditions on illness severity among adults hospitalized with COVID-19	COVID-19 can result in prolonged illness even among persons with milder outpatient illness, including young adults.
Andrew/2020 [29]	Report		Strong and coordinated surveillance and research focused on LTCFs and their frail residents is required. These efforts should include widespread assessment of frailty using feasible and readily implementable tools such as the CFS.
Wander/2023 [31]	Case-control study	ADL and IADL changes	Accurate and consistent documentation of U09.9 is needed to maximize its utility in tracking patients for clinical care and research
Ioannou/2022 [32]	Retrospective cohort study/198,601 SARS-CoV-2-positive persons	Rates, clinical setting, and factors associated with documented receipt of COVID-19-related care 3 or more months after acute infection (AOR, 1.71; 95% CI, 1.65–1.78)	COVID-19 vaccination has a potential protective effect against developing long COVID symptoms or manifestations.
Bull-Otterson/2022 [33]	Report		COVID-19 survivors have twice the risk for developing pulmonary embolism or respiratory conditions; one in five COVID-19 survivors aged 18–64 years and one in four survivors aged ≥65 years experienced at least one incident condition that might be attributable to previous COVID-19.
Cohen/2022 [35]	Retrospective cohort study/87,337 patients	The presence of persistent and new sequelae at 21 or more days after a diagnosis of COVID-19/HR 1.76 95% CI 1.58–1.97	Confirmation of an excess risk for persistent and new sequelae in adults aged ≥65 years after acute infection with SARS-CoV-2.
Mansell/2022 [37]	Personal view		Older people are the most affected when it comes to the effects of COVID-19. Long COVID must be considered in the differential diagnosis of symptoms that might otherwise be ascribed to frailty in older patients.
Hu/2023 [40]	Review		Vaccination before infection remains an essential measure in preventing long COVID, especially in elderly patients.
Giebel/2021 [42]	Survey	Impact of COVID-19 public health measures on access to social support services and the effects of closures of services on the mental well-being of older people and those affected by dementia	Being unable to access social support services due to COVID contributed to a worse quality of life and anxiety in those affected by dementia and older adults across the UK.

**Table 2:** Clinical features of long COVID in the older population.

First Author/Year	Study Design Sample Size	Population Outcome	Main Findings
Hayes/2021 [7]	Scoping review	Authors aggregated the type and prevalence of symptoms in people with long COVID	Most studies describe symptoms similar to those seen in acute COVID-19 infection, such as sensory impairment and respiratory symptoms. However, data indicates a broader range of symptoms, with over 100 reported symptoms. The prevalence of symptoms varied significantly and could not be attributed to data collection methods, study design, or other methodological factors, suggesting a potential link to unknown factors specific to the study cohorts.
Lopez-Leon/2021 [8]	Systematic review and meta-analysis	Identifying studies assessing the long-term effects of COVID-19 and estimating the prevalence of each symptom, sign, or laboratory parameter of patients at a post-COVID-19 stage	In the reviewed literature, are identified a total of 55 long-term effects associated with COVID-19. These effects primarily consist of clinical symptoms such as fatigue, headache, joint pain, anosmia, and ageusia, as well as diseases like stroke and diabetes mellitus. Additionally, measurable parameters included elevated levels of interleukin-6 (IL-6), procalcitonin, serum ferritin, C-reactive protein (CRP), N-terminal (NT)-pro hormone BNP (NT-proBNP), and D-dimer.
Madhavan/2021 [9]	Prospective cohort study	Estimating the prevalence, pattern, and functional outcomes of post-COVID-19 syndrome in hospitalized older adults	Post-COVID-19 syndrome affects approximately 9.3% of older adults, with fatigue being the most frequently reported symptom after 90 days of recovery, followed by cough and breathlessness.
Tosato/2021 [10]	Cross-sectional study	Determining the prevalence of persistent symptoms among older COVID-19 survivors and identifying symptom patterns.	Persistent symptoms are frequently experienced by older adults who have been hospitalized for COVID-19. Follow-up programs should be implemented to monitor and care for long-term COVID-19-related health issues.
Matsumoto/2023 [18]	Report	Summarizing the latest information on the impact of the pandemic on blood pressure control, the use of the renin-angiotensin system inhibitors in patients with COVID-19, and the blood pressure changes as one of the possible post-acute sequelae of COVID-19.	The evidence regarding the association between hypertension and the severity of COVID-19 is not clear, with mixed evidence available. To better understand the significance of hypertension in COVID-19, including the risk of blood pressure elevation in the post-acute phase, well-designed clinical trials are essential.
Xie/2022 [38]	Cohort study	Estimating risks and 1-year burdens of a set of pre-specified incident cardiovascular outcomes. HR 1.63 (95% CI—1.59, 1.68) of composite cardiovascular outcomes in patients with long COVID.	This study reveals that beyond the first month post-COVID-19 infection, individuals have a heightened likelihood of developing diverse cardiovascular conditions, including cerebrovascular disorders, dysrhythmias, ischemic and non-ischemic heart disease, pericarditis, myocarditis, heart failure, and thromboembolic disease.
Raman/2022 [43]	Review	Proposing a possible model for referral of post-COVID-19 patients to cardiac services and discussing future directions including research priorities and clinical trials	This review focuses on the concept of long COVID, discussing its definition and epidemiology, particularly emphasizing cardiopulmonary symptoms. It also delves into the pathophysiological mechanisms underlying both acute and chronic cardiovascular injury.
